# Bridging the Divide: A Review on the Implementation of Personalized Cancer Medicine

**DOI:** 10.3390/jpm14060561

**Published:** 2024-05-24

**Authors:** Michele Masucci, Claes Karlsson, Lennart Blomqvist, Ingemar Ernberg

**Affiliations:** 1 Department of Learning, Informatics, Management and Ethics (LIME), Karolinska Institutet, Tomtebodavägen 18B, 171 65 Solna, Sweden; 2 Department of Microbiology, Tumor and Cell Biology (MTC), Karolinska Institutet, Solnavägen 9, 171 65 Solna, Sweden; 3 Department of Oncology-Pathology (Onc-Pat), Karolinska Institutet, Anna Steckséns gata 30A, D2:04, 171 65 Solna, Sweden; claes.karlsson@ki.se; 4 Department of Molecular Medicine and Surgery (MMK), Karolinska Institutet, Anna Steckséns gata 53, 171 65 Solna, Sweden; lennart.k.blomqvist@ki.se

**Keywords:** individualized medicine, precision medicine, personalized medicine, stratified medicine, translational cancer research, cancer care, oncology, evidence-based medicine, team science, organization, management, scientific leadership

## Abstract

The shift towards personalized cancer medicine (PCM) represents a significant transformation in cancer care, emphasizing tailored treatments based on the genetic understanding of cancer at the cellular level. This review draws on recent literature to explore key factors influencing PCM implementation, highlighting the role of innovative leadership, interdisciplinary collaboration, and coordinated funding and regulatory strategies. Success in PCM relies on overcoming challenges such as integrating diverse medical disciplines, securing sustainable investment for shared infrastructures, and navigating complex regulatory landscapes. Effective leadership is crucial for fostering a culture of innovation and teamwork, essential for translating complex biological insights into personalized treatment strategies. The transition to PCM necessitates not only organizational adaptation but also the development of new professional roles and training programs, underscoring the need for a multidisciplinary approach and the importance of team science in overcoming the limitations of traditional medical paradigms. The conclusion underscores that PCM’s success hinges on creating collaborative environments that support innovation, adaptability, and shared vision among all stakeholders involved in cancer care.

## 1. Introduction

Cancer is a group of diseases with multiple causes and outcomes. Thus, the treatment of cancer, whether curative or palliative, employs a broad spectrum of methodologies. During recent decades, the molecular understanding of cancer has increased tremendously. The discovery of DNA structure as the genetic code [1] started a revolution in biomedicine that sees personalized medicine at the “harvest time” when the newly acquired knowledge of links between genes and diseases can be applied to diagnosis and treatment. In oncology, this paradigm shift has also provided the rationale for mechanism-based interventions that may complement or replace non-discriminatory methods, such as cytostatic drugs, radiation, and surgery, with procedures that are assumed to achieve an improved cure, cost efficacy, and reduced toxicity [2].

The molecular biology revolution has provided ample opportunities for discoveries resulting in new diagnostics and treatments [3]. The increased capacity and quality of sequencing technologies, combined with a dramatic reduction in costs, has fostered the emergence of new research areas that combine advanced cell biology, informatics, computer science, and medicine. At the same time, the extended biological knowledge made possible by scientific and technological advances has created the demand for new organizational structures, terminologies, and cultures in the translational space between research and healthcare.

The past two decades have seen an exponential growth in the number of scientific articles including “personalized medicine” in their title, with a total of 115,464 articles published until March 2024 and more than 103,198 published after 2010 (PubMed, March 2024). However, the increased popularity of the name does not correspond to a common understanding of the concept. Many attempts have been made towards a comprehensive definition of precision and personalized medicine [4]. A common reference is the paper by Schleidgen et al. [5] that reviews 2457 articles containing the term precision medicine. The authors argue that a common understanding of the definition of precision medicine is important for regulatory purposes. They posit that precision medicine is nothing new since medicine has always been individualized and tailored, providing a more technical understanding of precision medicine as the stratification of patients into treatment subgroups based on genetic or other omics-related information on the patient and disease. In his article Systems Biology and P4 Medicine: Past, Present, and Future [6], Hood expands the personalized concept within a systems medicine approach that combines predictive, preventive, personalized, and participatory perspectives into so-called “P4 medicine”. This comprehensive approach aspires to include multiple measurable biological markers and diagnostic methods in order to detect disease early and tailor treatments based on computational analysis of the specific patient dataset. According to The European Union’s Horizon 2020 Advisory Group, the concept of precision medicine can be described as “the characterization of individual phenotypes and genotypes (e.g., molecular profiling, medical imaging and lifestyle data) for tailoring the right therapeutic strategy for the right person at the right time, and/or to determine the predisposition to disease and/or to deliver timely and targeted prevention” [7].

A less comprehensive approach is implied in the concept of precision medicine that focuses on increased accuracy in diagnostic classification and treatment matching. Precision medicine strives to realise the medical principle “to provide the right treatment to the right patient at the right time” and has been particularly associated with the genomic stratification of patients into more exact disease subgroups. The concept has become a central focus of the expanding life science industry [8].

With continuous technical development, medical science is increasingly transformed from an intimate private practice between doctors and patients to a complex system comprising multiple disciplines, roles, and organizational structures. Achieving scientific discoveries is resource intensive, which results in a scientific culture concentrated on specific discoveries [9,10]. In parallel with concepts of precision, personalization, and individualization, which are oriented towards diagnostic and pharmaceutical aspects, the concepts of systems and networks have also been popularized. Systems biology has emerged as a popular conceptualization for the multiplicity of interrelated causal connections explaining macro phenomena such as cancer cell phenotypes. Networks, together with systems medicine, illustrate a form of medicine that cannot depend on the individual judgement of a single medical specialist but requires the concerted involvement of multiple experts that can provide different layers of information to the same patient case [11].

Despite multiple uses of the word “personalized” and associated terms, an established convention refers to “Personalization” as the capacity to tailor treatments based on the identification of molecular biomarkers that drive disease. While the explosive development of genomic technologies substantiates the hope that this concept may be rapidly translated into healthcare practices, the introduction of personalized medicine on a global scale is still a distant goal. Nevertheless, many actors have made considerable progress, which has provided a deeper understanding of what genomic and other biomolecular information can do to improve patient care. Their experience has given important insights into which forms of organization are better suited to manage a research and healthcare environment invested in personalized medicine [12].

## 2. Methodology

This review article involves an exploration of published scientific literature, focusing on identifying key concepts, theories, evidence sources, and research gaps related to the implementation of personalized cancer medicine. The guiding research questions were as follows: how is personalized medicine in cancer described in the scientific literature, and what are the determining factors for the introduction of personalized cancer medicine? To secure relevance, the headlines that developed for the review were vetted by a reference group of active professionals in the field.

A scoping review approach was selected for this study, which is ideal for exploring new research fields [13]. Given the diverse studies initially encountered, this method suited our goals well. The review followed the five-stage framework by Arksey and O’Malley [14] and adhered to the PRISMA scoping review guidelines for reporting [15]. Parts of articles were included for their capacity to describe central characteristics of personalized cancer medicine. Seminal articles known to the profession were included to provide context. However, the critical evaluation of medical findings in articles was not central, as our goal was to identify statements on organizational factors apart from the scientific significance and evidence. Additionally, there was no preregistered protocol for this review.

This review is based on published articles collected from five different sources: PubMed, Google Scholar, Research Gate, Web of Science, and Science Direct.

The following terms where searched for in whole articles, titles, and abstracts: “Personalized” OR “Personalised” OR “Precision” AND “Medicine” AND “Cancer” OR “Oncology” AND “Informatics” AND “Ethics” AND “Management” AND “Organisation” AND “Organization” AND “Translation” AND “Implementation”.

We adopted the following eligibility criteria for inclusion:

- Language: English.
- Published between January 2000 and April 2024.
- Published in a peer-reviewed journal.

**Figure 1:**
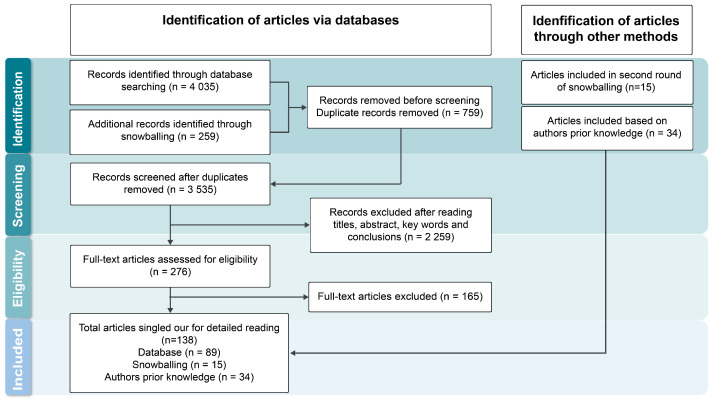
Flowchart of article selection process.

The search process initially identified 4035 unique records; an additional 259 records were added through snowball sampling. Of these, 759 duplicates where excluded. Altogether, 3535 articles underwent initial screening of titles, abstracts, keywords, and conclusions. The screening included 276 articles that were further examined in full to determine their eligibility and relevance. From this pool, 89 articles were singled out for detailed reading with textual descriptions. An additional 15 articles where identified from references and included based on relevance to emerging topics. A further 34 articles were chosen based on the authors’ prior knowledge. These articles where identified through association based on the categories that developed from the close reading of the first pool of 89 singled-out articles. Articles were selected based on relevance to describe key organizational factors for implementation or based on significance in describing personalized cancer medicine. Specific importance was given to sections of articles that disclosed contextual information on the conditions leading to the successful or failed translation of a specific method, treatment or other technology associated with personalized medicine, or helped to describe the field in a significant way. Interpretations and explanatory attempts were produced by exploring relationships between articles and concepts (Figure 1).

## 3. A Brief History of Personalized Cancer Medicine

The concept of PCM can be traced back to the mid-20th century when scientists first began to explore the relationship between genetics and cancer (Figure 1). Of course, the discovery of DNA as the genetic code in 1953 and subsequent developments to read the DNA base sequence started in 1977 were instrumental starting points for what can be done in molecular diagnostics today. However, PCM did not begin to develop until the 1990s, when new technologies such as large-scale DNA sequencing emerged. By the end of the 20th century, several complex causes of cancer could be described [16]. In the early 2000s, the completion of the Human Genome Project delivered a new research landscape, allowing scientists to identify specific genetic mutations that contribute to the development and progression of disease [17]. Early examples of this resource’s success include the discovery that certain lung cancers with an EGFR mutation respond to gefitinib therapy [18], the development of Her-2/Neu antibodies for breast cancer treatment [19], and the creation of the small molecule inhibitor imatinib, targeting the BRC/ABL fusion gene in chronic myelogenous leukemia [20]. However, in spite of the optimistic hope of quickly finding cures for all forms of cancer, the translation of rapidly increasing biological knowledge into efficient medicine has proven very challenging and time-consuming.

The US Food and Drug Administration (FDA) approved the use of imatinib (Gleevec®, manufactured and distributed by Novartis Pharmaceuticals, East Hanover, NJ, USA), the first cancer drug based on a patient’s genetic profile, in the early 2000s, marking a significant milestone in developing PCM [21]. Almost two decades later, in 2017, the use of pembrolizumab was approved by the FDA based on the presence of a genetic biomarker rather than the anatomical origin of the tumor [22]. Over the next decade, advances in omic technologies and big data analysis enabled the development of increasingly sophisticated methods for characterizing cancer at the molecular level, leading to the approval of many new targeted therapies for cancer (Table 1).

**Figure 2:**
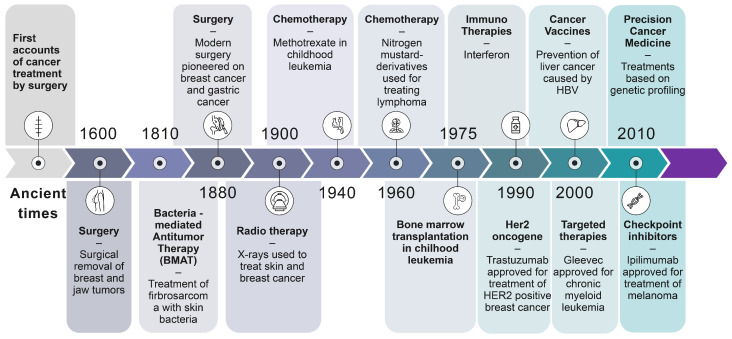
The history of cancer therapies. Sources: American Cancer Society, Cancer Research UK, and National Cancer Institute.

Today, “personalization” has become a cornerstone of cancer care, and many healthcare providers have integrated personalized approaches into their routine clinical practice with the hope of improving patient outcomes and quality of life. Despite these advances, much work remains to realize the potential of PCM (Figure 2).

## 4. The Challenges of Personalized Cancer Medicine

**Figure 3:**
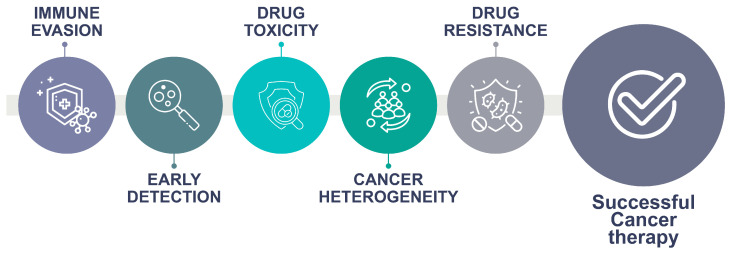
Cancer therapy faces a series of challenges to achieve successful outcomes.

The effective treatment of cancer faces many challenges. While early detection is crucial for effective cancer treatment, many cancers are not detected until they reach advanced stages [34]. The development of effective treatment is complicated by cancer heterogeneity, which refers to differences between individual cancers and even within different parts of the same tumor [35]. Medical oncologists are also faced with the enormous adaptability of cancer cells and their capacity to become resistant to treatment. Drug resistance is a common occurrence [36], which motivates the development of several alternative lines of treatment. A fundamental challenge within cancer is immune evasion [37], which entails the capacity of cancer cells to evade the immune system, making it difficult for the body to fight cancer effectively. Current trends in cancer treatment strategies are increasingly oriented towards the reactivation of the immune system’s ability to identify cancer cells as a means for a cure. Several successful studies have shown the potential of immune therapies [38], often in combination with conventional treatments. However, much still needs to be done regarding clinical effectiveness, especially in minimizing the risk of negative immune reactions with potentially lethal effects on patients. Finally, the ability to mitigate the toxicity of treatments [39] is still a major factor within all established cancer care plans. Many cancer treatments can cause significant side effects, making it difficult for patients to tolerate the treatments and thus limiting the treatment options due to patients’ performance status and other risk factors (Figure 3).

### 4.1. Achieving Precision within PCM

The effect of cancer heterogeneity on cancer treatments and personalized cancer medicine is significant because it can lead to treatment resistance, recurrence, and therapeutic failure [40,41]. To address these challenges, personalized cancer medicine aims to identify specific genetic and molecular changes in each patient’s tumor to inform the development of targeted and effective treatments. However, the effectiveness and actionability of PCM approaches, together with the complementary role of more conventional treatments, remain limited by cancer heterogeneity [42]. Thus, cancer heterogeneity represents a big paradigm change and a need for the continuous ongoing transformation of established treatment concepts primarily focused on the anatomic detection of cancer and the subsequent removal (surgery and radiotherapy) and killing of cancer cells (chemotherapy) [43].

### 4.2. Enabling Tools

Genomics is at the forefront of the precision medicine revolution and plays a fundamental role in today’s precision cancer medicine, offering comprehensive insights into the genomic alterations that drive tumor development, progression, and response to treatment [44]. Through the utilization of high-throughput genomic technologies like next-generation sequencing (NGS), researchers and clinicians are now equipped to employ personalized approaches for cancer patients. Genomic profiling has revolutionized cancer diagnosis and classification by surpassing the limitations of traditional histopathology methods [45]. By analyzing various genomic alterations, it enables more accurate characterization of tumors. The Cancer Genome Atlas (TCGA) project has been instrumental in enhancing our understanding of cancer subtypes and refining diagnosis based on genomic profiles [46]. Moreover, genomic profiling assists in distinguishing primary tumors from metastatic lesions, aiding in treatment decisions and identification of tumor origins. Genomic alterations also serve as valuable prognostic and predictive biomarkers, facilitating the development of personalized treatment strategies. By identifying these biomarkers through genomic profiling, clinicians can tailor treatment plans to individual patients. Targeted therapies that exploit cancer-associated genomic alterations have been made possible through genomic profiling, optimizing treatment selection while minimizing the use of non-targeted cytotoxic therapies. Liquid biopsies and genomic techniques for monitoring minimal residual disease offer non-invasive approaches to dynamically track tumor evolution and guide therapeutic interventions. Despite the rapid progress with new emerging omics technologies that will provide a more comprehensive spatial biological understanding of the tumour, genomics will continue to empower precision cancer medicine by providing a comprehensive understanding of tumor biology, aiding in diagnosis, treatment selection, and disease monitoring [47].

#### 4.2.1. Molecular Pathology

Molecular pathology of cancer refers to the use of molecular and genetic techniques to provide insights into the biology of cancer and create the conditions for informed, personalized cancer treatment. It involves the analysis of DNA, RNA, and proteins from cancer tissues and biopsy samples to identify specific genetic alterations, such as mutations and chromosomal rearrangements, that are significant factors for the emergence and progression of cancer [48,49].

#### 4.2.2. The Use of Imaging Modalities in Personalized Cancer Medicine

Imaging plays a crucial role in personalized cancer medicine by providing detailed visual information about a patient’s tumor and surrounding tissues. This information helps physicians determine the size, location, and extent of the cancer, as well as how it is affecting normal tissue. Computed tomography (CT), magnetic resonance imaging (MRI), and hybrid imaging using positron emission tomography (PET) combined with CT (PET/CT) are the main imaging modalities used for diagnostic purposes in oncology [50]. Advancements in precision oncology include the use of imaging for diagnosis, treatment planning, and monitoring response to therapy [51]. Several parallel imaging techniques are adopted throughout the cancer healthcare pathway. This includes ultrasonography, enabling real-time imaging for obtaining biopsy specimens and for directing local treatments. Routine surveillance of systemic treatment is usually performed by computed tomography (CT) as the first-line imaging modality. These modalities for cancer evaluation are increasingly complemented by molecular imaging techniques, which provide information specific to the tumor that indicates improvements in maneuverability and assessment of potential cancer treatments [52]. Furthermore, the systematization of imaging data using computational models, AI, and machine learning may further increase precision beyond subjective human assessment [53].

#### 4.2.3. Cellular Models for Drug Sensitivity Profiling

In recent years, the technology of using 3D cell cultures that can replicate the function of human organs has become an efficient tool in actionable cancer biology and precision medicine [54]. A live environment for cancer cells provides a more accurate model for studying cancer cell behavior, such as growth factors, drug reactions, and resistance. In personalized medicine, retrieving cancer cells from patients to test drug response has shown many promising results for several cancer types [55].

#### 4.2.4. Biobanking

The introduction of personalized cancer medicine has led to a shift in the way biobanking and biopsy procedures are performed and analyzed, with a greater focus on obtaining precise and informative samples and utilizing advanced technologies for analysis [56]. Biobanking involves collecting, storing, and managing biological samples (such as blood, tissue, or saliva) from patients with cancer. These samples are used to gather information about the patient’s tumor, including its genetic makeup, which can then be used for personalized treatment decisions [57]. The procedure is virtually the same as the introduction of personalized cancer medicine; however, it has had a significant impact on the way biopsies are performed and analyzed. In personalized cancer medicine, biopsies play a crucial role in providing information about the patient’s tumor and its genetic makeup, which is used for informed treatment decisions. Biopsies are now often performed with a greater level of precision, as they are guided by information gathered from genomic sequencing, imaging, and other tests. To maximize the amount of informative data, there is an increased interest in obtaining biopsy samples from specific parts of the tumor, such as areas with the highest levels of genetic abnormalities. In addition, the analysis of biopsy samples has become increasingly complemented by techniques such as NGS that allow comprehensive analysis of the patient’s tumor at the genetic level. With the introduction of fine-needle biopsies or fine-needle aspiration, access to tumor samples has been improved with fewer complications for patients [58]. This less invasive technique provides the opportunity for repeated biopsies, providing material for sequential and more precise diagnostics by following tumor progression, treatment effects, and possible resistance.

#### 4.2.5. Artificial Intelligence in Personalized Cancer Medicine

**Figure 4:**
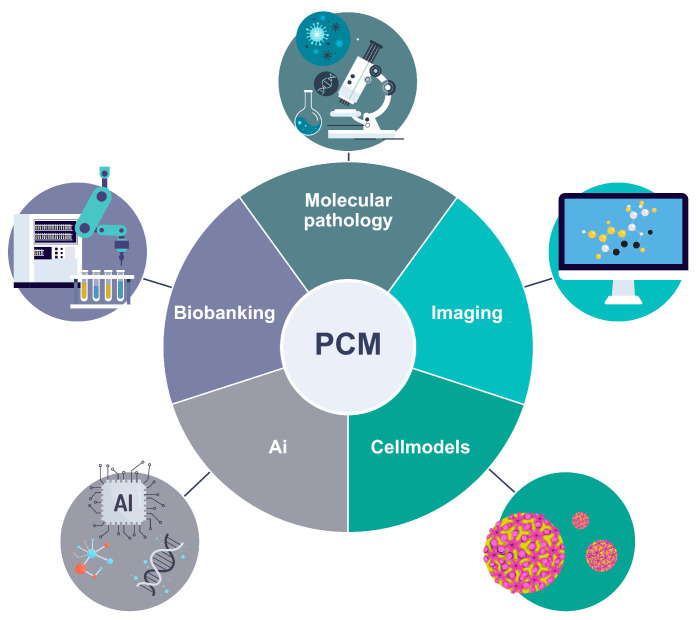
The main measures adopted within personalized cancer medicine.

The field of personalized cancer medicine is rapidly being transformed by emerging technologies based on artificial intelligence (AI) that are enabling a more efficient and accurate analysis of large amounts of data, including genomic data, imaging data, and other clinically applicable data sources with different modalities [59]. AI algorithms can help identify novel therapeutic targets and predict treatment responses, leading to more personalized treatment decisions for cancer patients [18,60,61,62] (Figure 4).

### 4.3. Implementations of Personalized Cancer Medicine

This chapter will provide a brief review of examples of how the concept of PCM has been implemented in clinical practice, including organizational insights from leading PCM studies.

Access to new treatments is a principal determining factor [63]. The possibility to access new, potentially more effective drugs and drug combinations is often described as a major obstacle in the implementation of precision medicine. Several authors mention organizational challenges as well as financial, legislative, and policy-related factors, making it difficult to design clinical drug studies for individually tailored combination therapies [64,65]. Proof-of-concept studies with single patients have demonstrated promising results and can inform the design of formal clinical trials [66]. According to Malani et al. [10], the use of a functional precision medicine tumor board (FPMTB) approach and the ex vivo drug response testing described in these studies can help establish international collaborations between public and private stakeholders to overcome current obstacles in the application of individually tailored precision medicine.

The advent of data-driven medicine, catalyzed by the introduction of systems medicine, has catalyzed a paradigm shift towards patient-centric cancer care. Despite the seeming tautology as all care and medicine are centered on benefiting the patient, patient involvement within the current complex translation scenario entails securing patient involvement on several parallel levels through different means. Patients now play an active role as agents in their treatment journey, contributing health data for early detection and intervention. Their involvement extends to clinical study design, enhancing research efficacy. However, challenges persist. Organizational hurdles impede the seamless integration of patient-generated data while privacy concerns loom. Moreover, while patient engagement holds potential, evidence gaps hinder informed implementation. The healthcare system must address these challenges to fully leverage personalized medicine’s benefits and empower patients effectively (Table 2).

## 5. Determining Factors in PCM

The majority of published scientific literature on personalized cancer medicine is focused on the revelation of significant positive results that provide insights into cancer biology and medical outcomes. Methodology sections and comment sections can include indirect comments on broader organizational, financial, legal, or even cultural determinants that the authors find significant for specific work or the field in general. These statements are important evidence of what the field itself perceives as significant determinants of the present conditions. In the article “Barriers and Facilitators to the Implementation of Personalised Medicine across Europe”, Stefanicka-Wojtas and Kurpas explore the needs, barriers, and facilitators of implementing personalized medicine (PM) in European healthcare systems [74]. The barriers for the implementation of precision medicine can be categorized into three main factors (policy, organizational, and economic) and six key stakeholders (patient, authorities, policy makers, funding agents, industry, and academia).

### 5.1. Health Policy Factors

The implementation of personalized medicine is resource-intensive and requires substantial changes in research and healthcare policy regulations [75]. Governmental support is vital for the success of paradigmatic change in healthcare. Health policies driven by governments impact the conditions for implementing personalized cancer medicine by ensuring that it is delivered in a responsible, ethical, and effective manner. Policies generally target patients’ access to healthcare, the conditions for the production and structure of high-quality healthcare and research data, patient rights, data privacy, and the regulation of the life science, pharmaceutical, and healthcare industries. Several government initiatives launched during recent decades have promoted personalized cancer medicine. In 2015, the US President Barack Obama made a State of the Union Address identifying personalized medicine as a prioritized area for healthcare (https://obamawhitehouse.archives.gov/precision-medicine (accessed on 5 May 2024)). The German Cancer Research Centre DKFZ (https://www.dkfz.de/en/index.html (accessed on 5 May 2024)) and Cancer Research UK (https://www.cancerresearchuk.org (accessed on 5 May 2024)) are other examples of large-scale national investments towards the introduction of personalized medicine in different fields. Other significant national initiatives are the Japanese genetic profiling multicenter study SCRUM-Japan MONSTAR-SCREEN-2 (https://www.scrum-japan.ncc.go.jp/monstar_screen/en/index.html (accessed on 5 May 2024)) and the European initiative Cancer Core Europé (https://cancercoreeurope.eu (accessed on 5 May 2024)). The US National Cancer Institute (NCI) initiated several programs to promote personalized cancer medicine, including the Precision Medicine Initiative (PMI) (https://www.cancer.gov/research/areas/treatment/pmi-oncology (accessed on 5 May 2024)) and the Cancer Genome Atlas (TCGA) (https://www.genome.gov/Funded-Programs-Projects/Cancer-Genome-Atlas (accessed on 5 May 2024)). In Europe, the Innovative Medicines Initiative (IMI) (https://www.imi.europa.eu (accessed on 5 May 2024)) is working to advance personalized medicine through research and the development of new diagnostic and treatment strategies. The UK’s National Health Service (NHS) is also a promoter of personalized cancer medicine through, for example, the NHS Cancer Plan and the 100,000 Genomes Project. The primary role of public healthcare policies should be to ensure that all patients have access to personalized cancer medicine, regardless of their financial status or location. Health insurance policies should provide coverage for personalized cancer medicine. Furthermore, before undergoing treatment, patients should be fully informed about the benefits and risks and provide their informed consent. Access to healthcare also encompasses that patients’ privacy and security can be guaranteed. At the same time, policies also need to support and fund ongoing research into personalized cancer medicine to ensure that it continues to advance and improve. Regulatory frameworks should both facilitate translational efforts and ensure that personalized cancer medicine is based on high-quality evidence and that its efficacy and safety are regularly monitored [76].

### 5.2. Health Economic Factors

The successful implementation of personalized cancer medicine is conditioned by several economic factors [77]. Developing a new medicine usually takes at least a decade and costs, on average, USD 2.6 billion from drug discovery to regulatory approval. Less than 12% of the candidate medicines make it to phase one clinical trials. Personalized cancer medicine often requires multiple funding agents, with traditional basic research funds not always geared towards application-centered research. Additionally, PCM frequently requires state or public support for infrastructures such as sequencing, clinical trial offices, molecular pathology, and IT. These barriers to PCM implementation have been documented in studies such as that of Chalmers et al. (2014) [78].

The costs associated with cancer care, including personalized cancer medicine, highlight the need for a careful balance between ensuring access to effective treatments and controlling healthcare costs [79]. This may require a combination of public and private investment, as well as a rethinking of traditional healthcare financing models. The relationship between public funding and industry investment is thus an important factor in understanding the introduction of personalized cancer medicine. Both public funding and industry investments have significant roles in the development of personalized cancer medicine. Public funding, often provided by government agencies such as the US National Cancer Institute, supports basic and clinical research into the underlying biology of cancer and the development of new drugs. This form of public funding provides the foundation for much of the innovation in the field and helps to advance the development of personalized cancer medicine. Industry investments, on the other hand, provide the financial resources needed to bring new personalized cancer treatments to market. Pharmaceutical and biotech companies are often the primary drivers of the development and commercialization of personalized cancer medicines, and their investments in research and development, clinical trials, and regulatory approval processes are essential for bringing these treatments to patients [80].

PCM faces several cost-related barriers, including expensive treatments and reimbursement policies that vary across countries and health insurance plans, potentially limiting patient access. The development of new PCM technologies requires significant investment, and companies may seek to recoup these costs through high prices for treatments. The adoption of PCM may also impact overall healthcare costs, as more individualized treatments may be more expensive than traditional treatments. The implementation of PCM requires the training of healthcare professionals, leading to additional costs, while the regulatory approval process can be lengthy and expensive, potentially impacting the availability of new treatments [81]. Cost-effectiveness in personalized cancer medicine refers to the cost–benefit balance of using personalized therapies compared to more traditional approaches [82]. This includes considering the cost of diagnostic tests, the cost of the personalized treatment itself, and any potential cost savings from improved patient outcomes and reduced side effects. In order to be considered cost-effective, the benefits of personalized cancer medicine, such as improved efficacy and reduced toxicity, must outweigh the additional costs incurred. Furthermore, cost-effectiveness analyses take into account the long-term benefits of personalized treatments for patients. This includes assessing the potential for reduced need for additional medicine and improved quality of life. The major objective of cost-effectiveness analyses is to balance the value of the treatment in terms of improved health outcomes for patients to its impact in terms of resource expenditure by the healthcare system and general society. Personalized medicine is estimated to have a significant potential to provide cost benefits in the treatment of cancer by enabling the development of targeted and more effective treatments [83]. For example, this can be done by increasing the evidence that certain treatments, such as expensive pharmaceuticals, have the desired effect and thus provide value to the patient. Personalized medicine has been shown to improve patient outcomes by providing more effective treatments and reducing the risk of side effects associated with chemotherapy and other traditional cancer treatments. This can lead to reduced costs associated with hospital stays and other medical procedures.

### 5.3. Organizational Factors

The translation of personalized medicine into clinical practice is dependent on several critical organizational factors. One critical factor is the need for a new form of scientific leadership. This includes researchers who are well-versed in the latest developments in the field and have experience in conducting clinical trials. Without this leadership, there may be a lack of direction and coordination, making it difficult to move research findings into clinical practice. Despite the challenges with assessing the effect of scientific leadership on scientific output in empirical terms, recent studies provide a clear indication of it as an important determining factor [84]. Another important factor is creating a clear communicative environment between all stakeholders involved in the translation process. Effective communication between researchers, clinicians, patients, and regulators is critical to ensure that everyone is working towards a common goal and that any challenges or obstacles are quickly addressed. Establishing conditions for team science is also critical. Personalized cancer medicine is a multidisciplinary field that requires collaboration between researchers with different areas of expertise, such as geneticists, oncologists, and pharmacologists [85]. A collaborative environment can foster innovation, allow for the exchange of ideas and expertise, and facilitate the development of more effective treatment options. Education is another critical factor in the translation of personalized cancer medicine. Physicians, researchers, and other healthcare professionals need to be well-educated in the latest developments in the field to ensure that they can effectively diagnose and treat patients. Additionally, educating patients about personalized cancer medicine and its potential benefits can help to increase patient engagement and support for research efforts.

Efforts to boost translation from public and private interests are essential. Collaboration between biotech and pharmaceutical companies, academic institutions, and government regulators is essential to accelerate the translation of personalized cancer medicine into clinical practice. Additionally, targeted investment in research infrastructure and the development of new technologies can help to speed up the translation process. Regulation and government policy play a significant role in the translation of personalized cancer medicine. Regulations related to clinical trials, drug development, and approval processes can impact the translation of research findings into clinical practice. Government policies related to funding and investment in research infrastructure can also influence the translation process.

### 5.4. Impact of the National Organization of University-Associated Health Care

The prerequisites for the introduction of PCM show large variations between countries [86]. These differences are dominated by three factors: the extent of government involvement and common funding initiatives, as already discussed, the organization of clinical research and university-associated health care, and the organization of healthcare insurance [87,88]. The institutions spearheading the advancement of personalized cancer medicine are mainly university hospitals and their associated medical faculties dedicated to translational cancer research. These organizations range from comprehensive cancer centers that integrate basic and clinical research with cancer care under unified leadership to federal medical universities that collaborate with regional hospitals under separate leaderships in countries like Germany, Sweden, and the UK. The former ones are known as “cancer centers”, these institutions may operate as private entities or foundations (such as MD Anderson, Mayo Clinic, and Memorial Sloan Kettering in the US, and VHIO in Barcelona) or as public institutions (such as Gustave Roussy in France and the Netherlands Cancer Institute). Centers with unified leadership in research and cancer care tend to be more agile and flexible and benefit from the reallocation of resources between their primary activities, for example, using income from patient fees to support translational research.

There is also a significant difference in how healthcare insurance systems affect funding for new drugs and treatment combinations, depending on whether they are distributed and/or largely private (as in the US, Germany, and the Netherlands) or publicly financed through taxes (as in Scandinavia and the UK). In systems that are distributed and private, it is often easier to negotiate funding for the introduction of new targeted drugs [89,90].

## 6. Implementing PCM

The introduction of PCM should ensure that patients receive the best possible care while also taking into account the needs and interests of different stakeholders, including healthcare providers, policymakers, and industries. This requires careful planning and coordination to ensure that the approach is effective, equitable, and sustainable.

### 6.1. PCM Requires Scientific Leadership

Several sources emphasize that personalized cancer medicine requires strong scientific leadership to guide research, development, and implementation [91]. One of the primary roles of scientific leadership is to set the research agenda. Scientific leaders must identify the most pressing questions and challenges in the field and prioritize the allocation of resources towards finding solutions. This requires a deep understanding of the latest advances in the field and the ability to identify areas where further research is needed. Scientific leaders are also required to create the ideal conditions to facilitate development. They must work closely with clinicians, researchers, and technology developers to translate scientific discoveries into practical applications. This requires strong communication and collaboration skills, as well as an understanding of the complex regulatory and ethical issues involved in personalized medicine. Scientific leadership is crucial in setting up routines and procedures that can guarantee standards, such as high quality, accessibility, and reliability of the data that forms the basis of PCM.

Numerous studies have explored the impact of scientific leadership on the successful introduction of personalized cancer medicine [92]. For example, Luchini et al. [59] found that strong scientific leadership was critical for the development of new AI-based technologies in cancer diagnosis and treatment. Abernethy et al. (2010) [93] posit that political and scientific leadership working closely with clinicians, researchers, and technology developers ensure the rapid translation of scientific findings through “Rapid-learning systems”, providing actionable and practical applications that can improve treatment outcomes.

### 6.2. PCM Depends on Team Science

The systemic character of precision medicine requires collaboration across a multitude of different professions and organizations [85]. The establishment of interdisciplinary collaborations between biomedical researchers, clinical expertise, informatics, and computational expertise has brought about new titles and medical professions that are complementing or, in some cases, replacing old categories. The difference in scope, funding, regulation, and organizational culture between science and healthcare provokes a series of challenges. Conventional scientific literature lacks or provides minimal insights into the organizational measures needed to transform organizations towards a personalized medicine capacity. Translational cancer research is one of the fields that depend on team science, a discipline that aims to solve complex scientific challenges and build adequate organizations or infrastructures [94]. The focus of team science is to bring together experts from different fields to work together and leverage their collective expertise to tackle issues that cannot be addressed by individual researchers alone. Team science recognizes that complex problems require interdisciplinary approaches and that diverse perspectives are essential to the development of new knowledge and innovative solutions. Effective team science involves clear communication, shared decision-making, and a supportive, collaborative work environment that fosters creativity, productivity, and innovation.

### 6.3. Work Conditions Are a Key Implementation Factor

Work conditions play a significant role in successful translation. Optimal work conditions are determined by access to adequate funding, technology, and a supportive organizational structure and culture. By addressing these factors, organizations can improve the quality of care and advance the field of personalized medicine in cancer. Clinical researchers and nurses have a fundamental role in the implementation of personalized cancer medicine. Work conditions for clinical researchers and nurses, such as job satisfaction, workload, and resources, can affect performance and, ultimately, the capacity for translation [95]. The quality of their work and their possibility to operate under good conditions directly affects patient outcomes [96]. A common issue is high workloads and stress levels over long periods of time. This can lead to burnout among clinical researchers and nurses, which can negatively impact their ability to provide quality care to patients [97]. Inadequate funding or inadequate staffing can impact the ability of clinical researchers and nurses to carry out research activities to prioritize essential care activities, stalling an effective implementation of personalized cancer medicine. Access to state-of-the-art equipment and technology is also essential for ensuring that researchers and clinicians have the tools they need to effectively diagnose and treat cancer.

Another aspect of work conditions that can impact the successful translation is the organizational structure and culture of the workplace [98]. For example, a hierarchical structure can create barriers to communication and collaboration, while a culture that values innovation and interdisciplinary collaboration can support the development and implementation of personalized medicine. The culture of the workplace defined by a multitude of factors can also impact the successful translation of personalized medicine in cancer [99].

### 6.4. Enabling Collaboration between Industry and Academia

The successful translation of personalized medicine in cancer is highly dependent on good relationships between industry and academia in order to close the gap between basic scientific research and concretely actionable care in the clinic [100]. While academic researchers explore new scientific concepts and innovative technologies, the industry focuses on the commercialization of these technologies and the development of new products [101]. These collaborations can also help to overcome barriers of commercialization, such as the high cost associated with research, including adhering to a complex regulatory environment. Moreover, industry–academia collaborations are essential for the development of new technologies, such as AI-based technologies, diagnostic methods, or new drugs. Industry–academia collaborations can bring together the expertise and resources of both sectors, leading to the development of new predictive models and the testing, transfer, and implementation of scientific discoveries into clinical practice [102].

**Figure 5:**
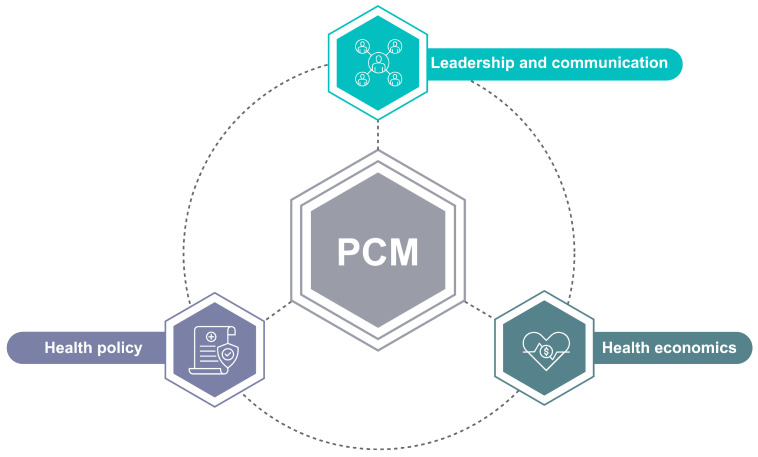
Implementing personalized cancer medicine comprises several organizational challenges.

The relationship between industry and academia has been explored in several scientific articles studying personalized medicine in cancer, but there is no consensus on the optimal models for implementation (Bornstein et al., 2011) [103,104,105]. In France and the UK, academic hospitals provide genomic testing free of charge, whereas, in countries like the US, biomarker companies offer these as paid services. It is yet to be defined what the optimal model for implementation should be and how it should align with the overall model of the healthcare system [106] (Figure 5).

## 7. Addressing the Translational Divide: Enabling Technology and Innovation Transfer from Biomedical Research to Diagnosis and Treatment

### 7.1. Addressing Legal and Ethical Issues

Several legal and ethical issues related to the implementation of PCM are discussed in the literature. Personalized cancer medicine follows the general trend within advanced modern medicine of increased reliance on the collection and analysis of sensitive personal and medical data. This essential activity raises concerns about data privacy and security for patients. To achieve the best possible care in the PCM context, legal issues need careful consideration to ensure that patients receive optimal care while protecting their rights and interests. The legal costs associated with these challenges can vary widely and may include fees for legal advice, patent applications, regulatory submissions, and litigation of any disputes that arise. Personalized cancer medicine often involves complex molecular and genetic testing, which can lead to disputes over who owns the data and any resulting treatments. The development of personalized cancer treatments entails significant investment in research and development, leading to questions about ownership of the rights to the resulting diagnostic tests, treatments, and technologies. Personalized cancer medicine generates large amounts of sensitive personal information, which must be protected under privacy laws. Companies that develop and market personalized cancer medicines may face liability for any adverse events or lack of efficacy associated with the treatment. Personalized cancer medicine may involve the use of experimental treatments, which raises questions about liability and informed consent, particularly if the treatment is not effective or causes harm. The development and approval of personalized cancer treatments are subject to rigorous regulatory standards, which can impact the speed and availability of these treatments [107]. Finally, personalized cancer medicine can be more expensive than traditional treatments, which raises questions about reimbursement by insurance companies and government healthcare programs.

The implementation of PCM brings to the forefront several ethical issues that must be considered [108]. One of the primary ethical concerns is access to care [109]. Since personalized cancer medicine is usually expensive, not all patients may have equal access to these treatments, raising questions of fairness and equality. Data privacy is another ethical issue. The collection and computation of large amounts of complex and heterogeneous patient data raise concerns about privacy and confidentiality. The risk of discrimination is another ethical concern. Unequal treatment based on factors such as race, gender, or socioeconomic status could result in discrimination. Informed consent is an essential component of ethical medical practice, and personalized cancer medicine is no exception. An important ethical issue is clinical validity. Since personalized cancer medicine is still in the early stages of development, the validity and reliability of these treatments may be questionable, leading to ethical concerns about the use of unproven or ineffective treatments [110].

Informed consent is an essential component of ethical medical practice, and personalized cancer medicine is no exception. Patients need to be fully informed about the benefits and risks of personalized cancer medicine and must provide their informed consent before treatment begins. Moreover, conflicts of interest are a common ethical dilemma in personalized cancer medicine. The financial interests of pharmaceutical companies and healthcare providers may conflict with the best interests of patients, leading to ethical dilemmas. Addressing these ethical concerns is critical to ensuring that personalized cancer medicine is implemented in a responsible and ethical manner. To achieve this goal, policymakers, researchers, and healthcare providers must work together to develop ethical guidelines for personalized cancer medicine that consider the perspectives of patients, healthcare providers, and society as a whole.

In recent years, the EU has been following a development in the North American context regarding stricter requirements on quality and standards for medical devices and wet lab tests used for patient care. In 2022, the In Vitro Diagnostic Regulation (IVDR) replaced an older directive, which will greatly impact the processes of introducing advanced and large-scale molecular diagnostics in the clinical setting.

### 7.2. Achieving Equality for Personalized Cancer Medicine

Achieving equality in personalized cancer medicine can be a challenge, as access to these treatments is often limited by factors such as cost, availability, and geographic location [111]. Several strategies can be employed to help ensure that everyone has access to personalized cancer treatments, regardless of their ability to pay or where they live [112]. Achieving equality in personalized cancer treatments requires that everyone has access to essential health services, including through universal health coverage programs. Universal health coverage ensures that everyone has access to healthcare services. This is particularly important for individuals who are unable to pay for the cost of these treatments or have other barriers to access [113]. Patient assistance programs, either through government programs or charitable organizations, can provide financial assistance to these individuals.

Community-based initiatives can also play a critical role in ensuring equality in personalized cancer treatments [114]. Working with local communities to raise awareness of personalized cancer medicine can help to remove barriers to access and improve health outcomes. Community-based initiatives can also provide access to these treatments to those who might not otherwise have access. This may involve increasing the availability of diagnostic tests in low- and middle-income countries, where access to these tests may be unavailable [115].

To ensure that everyone has equal and fair access to personalized cancer treatments, productive collaborative conditions must exist between healthcare providers and patients. This involves providing patients with the relevant information and resources needed for qualified medical decisions. Access to information and resources can be improved through the development of productive collaborative conditions, which enable patients to work with healthcare providers to identify the most appropriate treatments for their individual needs. By addressing these key factors, we can work towards achieving equality in personalized cancer treatments [116].

In summary, the available literature suggests that the following actions are required for the successful introduction of personalized cancer medicine into clinical practice:

- Engage with key stakeholders, including patients, healthcare providers, policymakers, and industries, to ensure that the introduction of personalized cancer medicine is informed by the needs and perspectives of all those who will be affected.
- Conduct rigorous clinical validation studies to conclusively demonstrate how personalized cancer treatments are efficient and safe in conjunction with their impact on patient outcomes.
- Work with regulators and payers to ensure that personalized cancer treatments are approved for use and covered by insurance (public or private) so that patients have access to these treatments when they need them.
- Provide education and training for healthcare providers and patients to ensure that everyone has the information and resources they require to make well-informed decisions about personalized cancer medicine.
- Develop and implement effective data management policies and procedures to ensure that patient data is handled appropriately and securely.
- Address the issue of equality and access to ensure that everyone has access to personalized cancer treatments regardless of the patients’ ability to pay, social status, or residence.
- Continuously evaluate and improve the introduction of personalized cancer medicine to ensure that patients receive the best possible care and that the approach remains effective over time.

### 7.3. Education and Dissemination

An often-mentioned key factor for succeeding in implementing the paradigmatic shift into personalized medicine is the preparation of new generations of medical professionals. Part of medical professionals’ resistance to new medical concepts can be addressed with a change-in-management approach [117]. However, a complete transformation might only be possible once the new generations take on leadership positions. More importantly, given the rapid developments in the field, there will be a need for efficient dissemination and further education of persons with key roles in the intertwined chain of translation.

### 7.4. Patient Involvement

Active patient involvement, participation, and agency are vital components of the concept of personalization, extending to related concepts such as P4 medicine, which emphasizes “participation” [118]. Among these, perhaps the most pivotal aspect is the patient’s consent to participate in clinical trials or, more fundamentally, to accept recommended medical treatment. However, in recent years, the involvement of patients in various stages of treatment has emerged as a field of extensive research and development [119]. Patients should not be perceived merely as passive recipients of medicine or dehumanized sources of data for research purposes. While the primary goal of medicine is to enhance patients’ lives and health, this fundamental aim can sometimes be overshadowed by the intricate network of efforts spanning from research to healthcare delivery. Integrating patients’ expressed ideas, intuitions, and self-reported symptoms can provide a unique source of information to complement the multitude of biological markers informing diagnosis and treatment decisions [120]. Furthermore, granting agency to patients leads to a genuinely personalized healthcare experience. Transforming the traditionally passive and subordinate role of patients into a more active and rewarding experience preserves patients’ integrity and dignity. Although patients should not feel obligated or pressured to delve into the complexities of their medical conditions and treatments, fostering an environment conducive to patient involvement is crucial for enhancing their relationship with healthcare. Ongoing studies demonstrate the value of structured patient-reported symptoms, offering critical insights to guide physicians during pivotal decision-making moments. Additionally, patient-reported symptoms and outcomes serve as valuable predictive markers, enhancing the precision of dosing and treatment protocols [121,122].

## 8. New Forms of Organization That Bridge the Divide

**Figure 6:**
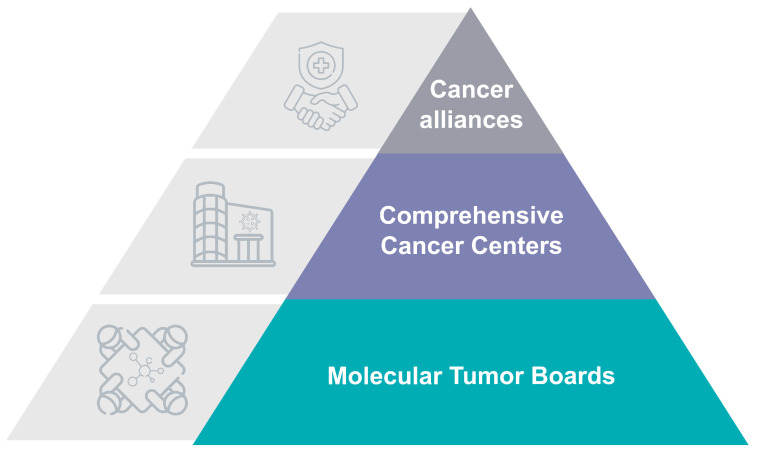
New forms of organization to bridge the divide.

The implementation of PCM has fostered the building of new organizations capable of handling the complexity of a rapidly developing discipline (Figure 6).

### 8.1. Molecular Tumor Boards

The molecular tumor board (MTB) is an organizational innovation that has emerged from the introduction of personalized medicine approaches to cancer care [123,124,125]. With the introduction of molecular markers, the increased complexity of tumor classification and treatment selection has urged oncologists to form specific meetings where actionable targets are discussed based on results from genomic analysis and other available evidence [126]. MTBs are multidisciplinary teams of experts from different fields, including oncology, genetics, pathology, and bioinformatics, that are brought together regularly to analyze large amounts of genomic data and translate this information into treatment recommendations based on the molecular characteristics of patient’s tumors and decisions on potential inclusion to clinical trials [127]. The introduction of these meetings has affected the translational and implementation processes for personalized cancer medicine [128]. MTBs are expected to become the perfect translational means by which to extend molecular diagnostic tools, including multi-omics with transcriptomics and proteomics [129].

Several studies have demonstrated the impact of MTB on the implementation processes for personalized cancer medicine [123]. MTBs have been shown to improve the accuracy of genomic data interpretation and increase the proportion of patients who received targeted therapy [130,131,132]. This also includes the improvement of the capacity to communicate with patients by facilitating the presentation of genomic information [133]. This has improved the adoption of precision medicine in cancer care and helped to accelerate the translation of genomic discoveries into clinical practice. MTBs also provide a framework for educating clinicians and guiding them through incorporating genomic data into clinical decision-making [131]. The organizational phenomena of MTBs are becoming more institutionalized with the introduction of transnational cooperation and studies investigating the procedures and results of these meetings. Furthermore, tools such as decision support systems designed to improve the quality of decisions during MTBs by generating digestible data overviews based on large quantities of accumulated evidence have been proven effective [134].

### 8.2. Comprehensive Cancer Centers

Comprehensive cancer centers (CCCs) have been identified as the main catalyzers to bridge the translational divide. CCCs are specialized institutions at the level of medical faculties and university hospitals or cancer centers that aim to provide a seamless integration of cancer research and patient care [135,136].

They are designed to bridge the divide between research and the clinic by creating an environment where scientists and clinicians can work together to develop and implement new cancer treatments. In this environment, a unique approach to cancer care is provided by bringing together medical professionals, including oncologists, surgeons, radiologists, pathologists, and nurses, with biomedical researchers, to provide a comprehensive and personalized treatment plan for each patient following team science principles.

A key feature of CCCs is the emphasis on multidisciplinarity. By bringing together experts from different fields, CCCs can provide a more holistic approach to cancer care, taking into account not only the medical aspects but also the social, psychological, and emotional needs of patients. Tumor management groups are a common example of multidisciplinary teams, where different specialists collaborate to determine the most effective treatment plan for each patient.

The success of CCCs is critically dependent on their organization. Program directors are typically scientists who are responsible for fostering interactions between different institutes and programs within the CCC. These interactions can be informal, such as regular meetings or collaborations, or more formal, such as sponsored symposia or workshops. By creating a collaborative environment, CCCs encourage the sharing of knowledge and expertise, leading to more efficient and effective cancer treatments. The functionality of relevant branches is essential to the CCC’s operation. CCCs must have the necessary infrastructure, equipment, and personnel to carry out cancer research and clinical trials. These resources are typically shared among different programs and divisions within the CCC, ensuring that all researchers and clinicians have access to the tools they need to advance their work.

The establishment of comprehensive care networks is another crucial aspect of CCCs’ operation. These networks connect the CCC with other healthcare providers in the region, such as community hospitals and primary care physicians. By collaborating with these providers, CCCs can ensure seamless transitions between different levels of care, providing patients with the best possible treatment at every stage of their cancer journey [135].

Governance is another critical component of CCCs. These institutions are typically governed by a board of directors, which oversees the operations of the CCC and ensures that it is meeting its goals and objectives. The board is responsible for ensuring that the CCC has the necessary resources to carry out its mission and that it is adhering to ethical and legal standards.

### 8.3. Large-Scale Infrastructure and Screening Consortia

In the realm of precision medicine in cancer, large-scale infrastructures play a vital role from an organizational perspective. These infrastructures encompass various interconnected elements such as data integration platforms, high-performance computing systems, advanced analytics tools, and collaborative networks. By establishing such infrastructures, organizations can effectively harness the vast amounts of genomic, clinical, and imaging data that are generated in cancer research and treatment. These integrated systems facilitate seamless data sharing, storage, and analysis, thereby empowering researchers and clinicians to gain deeper insights into the molecular underpinnings of cancer and develop personalized treatment approaches. A notable example is the work by Alarcon Garavito et al. (2023) [137], which showcases the successful implementation of a large-scale infrastructure that integrates genomic data, electronic health records, and computational resources to bolster precision medicine endeavors in the field of oncology. This infrastructure not only expedites the identification of actionable genomic alterations but also provides real-time support for clinical decision-making, ultimately leading to improved patient outcomes (Table 3). In the European context, several large-scale national infrastructures are worth mentioning, such as Science for Life Laboratory and Genomics Medicine Sweden [138], and Genomics Medicine England [139].

## 9. Conclusions

Herein, we emphasize the need for a comprehensive and systematic qualitative study on the determining factors for the successful translation of personalized cancer medicine. Despite the methodological challenges associated with an exhaustive analysis (addressing social complexity, contextual difference, and biases) of the conditions required for the successful implementation of translational approaches, a number of factors can still be identified as significant. PCM faces several challenges that need to be addressed, including funding, infrastructure support, and the development of new regulatory approval processes. Overcoming these challenges will require multi-stakeholder collaboration, including governments, healthcare systems, research institutions, and the pharmaceutical industry, to ensure that patients can access the benefits of personalized cancer medicine. In addition, the translation of personalized cancer medicine into clinical practice requires the convergence of various environmental factors, including strong scientific leadership, a clear communicative environment, and the establishment of collaborative team science. Matching science and education with patients’ needs and regulatory policies plays a critical role in the translation process. Effective data management is essential to ensure the success of personalized cancer medicine, and careful consideration and planning are required to achieve this aspect. In-house infrastructure needs are essential, as they can help to ensure that patient data are managed appropriately and securely. The capacity to build relationships within the industry is important, as it can help to foster collaborations and facilitate the development and implementation of new technologies. Beyond the fragmentation in fostering bridging connections, a recurring observation from multiple stakeholders is the fragmented nature of the relationship between research, healthcare, industry, government, and patients [140]. There are multiple obstructions, or lack of capacities, resulting in divides in the continuum from research to healthcare. Interviews with stakeholders have confirmed this fragmentation, which is resulting in different obstructions in translational efforts. Possible measures to bridge the divides include the following:

- Pooling resources across stakeholders;
- Investing in time for clinical investigators to provide the best working conditions so they can move the translational process forward;
- Providing the right conditions for a multitude of clinical trials of different designs, sponsorships, and purposes.

Pooling common resources across stakeholders is essential for advancing translational research in medicine. Bringing together researchers, healthcare providers, and other stakeholders, can leverage the collective expertise and resources to accelerate the development of new treatments and therapies. However, it is not just about having access to resources but also investing in time to ensure that clinical investigators have the best working conditions to bring the translational process forward. This includes providing the necessary infrastructure, support staff, and funding to carry out clinical trials of different designs, sponsorships, and purposes. By creating the conditions for a multitude of clinical trials, one can improve the efficiency and effectiveness of the translational process, leading to better outcomes for patients.

## Figures and Tables

**Table 1:** The development of current established targeted therapies in cancer based on the date on which pioneering articles were published.

Targeted Therapies	Pioneering Articles	Main Indicators
HER2-Target Therapies	(Slamon et al., 2000) [19]	Breast cancer
EGFR Inhibitors	(Lynch et al., 2004) [23]	Non-small cell lung cancer and colorectal cancer
MEK Inhibitors	(Adjei et al., 2008) [24]	Melanoma with BRAF mutations and non-small cell lung cancer
FGFR Inhibitors	(Fischer et al., 2008) [25]	Urothelial bladder cancer with FGFR alterations and cholangiocarcinoma with FGRFR alterations
PARP Inhibitors	(Fong et al., 2010) [26]	Ovarian cancer, breast cancer, and pancreatic cancer with BRCA mutations
BRAF Inhibitors	(Flaherty et al., 2010) [27]	Melanoma with BRAF mutations; colorectal cancer with BRAF mutations
ALK Inhibitors	(Kwak et al., 2010) [28]	Non-small cell lung cancer
BRD4 Inhibitors	(Delmore et al., 2011) [29]	No specific cancer
ROS1 Inhibitors	(Shaw et al., 2014) [30]	Non-small cell lung cancer
CDK4/6 Inhibitors	(Finn et al., 2016) [31]	HER2-negative breast cancer
RET Inhibitors	(Drilon et al., 2020) [32]	Medullary thyroid cancer with RET mutations; lung cancer with RET rearrangements
TRK Inhibitors	(Drilon et al., 2020) [32]	Cancers with NTRK gene fusions, various cancer types, pediatric cancers, and solid tumors
KRAS Inhibitors	(Hong et al., 2020) [33]	Colorectal cancer and lung cancer

**Table 2:** Organizational insights from leading PCM studies. The most prominent PCM-characterized clinical trials bring both scientific and organizational insights. This table singles out some key observations based on the clinical trial design and organizational model selected for the study from a selection of published papers from the studies.

PCM Study	Selected Organizational Insights
ComboMATCH [67] (Meric-Bernstam et al., 2023)	ComboMATCH, a precision medicine initiative, is developed collaboratively with NCI/CTEP and academia, fostering interdisciplinary engagement. It leverages the CTEP Investigational New Drug program, integrating resources like ETCTN and PDXNet. This patient-centric approach utilizes routine clinical genomic profiling for therapy selection, emphasizing teamwork, and biomarker-directed therapy to enhance patient outcomes.
DRUP, 2023 [68] (Geurts et al., 2023)	Drug repurposing and biomarker-driven treatment selection, which required collaboration with pharmaceutical companies to obtain access to the drugs and to ensure that they were provided to patients in a timely and consistent manner.
MASTER, 2021 [69] (Cecchini et al., 2019)	Algorithm-based treatment selection and comprehensive molecular profiling with a multidisciplinary team approach. Showcasing the potential of algorithm-based treatment selection and comprehensive molecular profiling.
Mi-ONCOSEQ, 2021 [70] (King et al., 2021)	Comprehensive genomic profiling using a multidisciplinary team approach. Need for a coordinated effort between multiple centers to achieve a sufficient sample size.
WINTHER, 2019 [71] (Rodon et al., 2019)	A novel “window of opportunity” design. Coordinated effort required between multiple centers to achieve a sufficient sample size.
MOSCATO, 2017 [72] (Massard et al., 2017)	Emphasis on collaboration but extensive coordination is required. Importance of data sharing and collaboration in precision medicine research.
SHIVA, 2015 [73] (Le Tourneau et al., 2015)	Importance of a coordinated, multidisciplinary approach to clinical research.

**Table 3:** National cancer initiatives promoting precision in cancer medicine.

Consortia	Start Year	Annual Budget
Genomics Medicine Sweden	2019	EUR 81 million
Cancer Research UK: International Cancer	2008	EUR 210 million
National Cancer Institute: Cancer Research	2005	EUR 4.6 billion
Genomics Medicine England	2013	EUR 123 million

## Data Availability

Copies of data are available upon request.
